# Feasibility and Clinical Value of CT-Guided ^125^I Brachytherapy for Pain Palliation in Patients With Breast Cancer and Bone Metastases After External Beam Radiotherapy Failure

**DOI:** 10.3389/fonc.2021.627158

**Published:** 2021-03-05

**Authors:** Jian He, Qicong Mai, Fangfang Yang, Wenhang Zhuang, Qing Gou, Zejian Zhou, Rongde Xu, Xiaoming Chen, Zhiqiang Mo

**Affiliations:** ^1^Department of Interventional Radiology, Guangdong Provincial People's Hospital, Guangdong Academy of Medical Sciences, Guangzhou, China; ^2^Department of Medical Simulation Center, Guangdong Provincial People's Hospital, Guangdong Academy of Medical Sciences, Guangzhou, China

**Keywords:** iodine-125, brachytherapy, palliative medicine, bone neoplasms, breast neoplasms

## Abstract

**Objectives:** To evaluate the feasibility and clinical value of CT-guided iodine-125 (^125^I) brachytherapy for pain palliation in patients with breast cancer and bone metastases after external beam radiotherapy failure.

**Methods:** From January 2014 to July 2016, a total of 90 patients, who had received the standard therapies for bone metastases but still suffered moderate-to-severe pain, were retrospectively studied. About 42 patients were treated with both ^125^I brachytherapy and bisphosphonates (Group A), and 48 patients were treated with bisphosphonates alone (Group B).

**Results:** In Group A, 45 ^125^I brachytherapy procedures were performed in 42 patients with 69 bone metastases; the primary success rate of ^125^I seed implantation was 92.9%, without severe complications. Regarding pain progression of the two groups, Group A exhibited significant relief in “worst pain,” “least pain,” “average pain,” and “present pain” 3-day after treatment and could achieve a 12-week-remission for “worst pain,” “least pain,” “average pain,” and “present pain.” The morphine-equivalent 24-h analgesic dose at 3 days, 4 weeks, 8 weeks, and 12 weeks was 91 ± 27, 53 ± 13, 31 ± 17, and 34 ± 12 mg for Group A, and 129 ± 21, 61 ± 16, 53 ± 15, and 105 ± 23 mg for Group B. Group A experienced a lower incidence of analgesic-related adverse events and better quality of life than Group B.

**Conclusion:** The CT-guided ^125^I brachytherapy is a feasible and an effective treatment for the palliation of pain caused by bone metastases from breast cancer after external beam radiotherapy failure.

## Introduction

Approximately 65–75% of patients with advanced breast cancer develop bone metastases (1), which may cause skeletal-related events (SREs), such as bone pain, pathological fracture, and hypercalcemia (2). Of these events, bone pain is the earliest symptom that conspicuously decreases the quality of life of patients (1). Thus, pain palliation is the primary therapeutic goal in the management of bone metastases.

Chemotherapy and endocrine therapy are the currently used common baseline treatments for patients with advanced breast cancer and bone metastases (3). Nevertheless, previous studies have shown that such systemic therapies achieve limited long-term bone pain relief (4). Consequently, the loco-regional treatment is used to supplement systemic therapies. The external beam radiation therapy (EBRT) is the most effective local treatment for pain palliation, reportedly achieving remission in up to 60% of patients (2). In some patients, the prescribed the EBRT dose is limited when the bone metastases are adjacent to vital organs such as the spinal cord (5); therefore, the pain relief effect could be compromised. For patients with relapsing pain, bisphosphonates in combination with analgesics are needed to manage the response of the body to the pain. However, when patients receive high dosages of analgesics for pain control, dose-related adverse effects increase significantly, and the quality of life of patients is not improved (1). Radiofrequency ablation effectively and promptly achieves pain palliation in patients with bone tumors (6). However, the ablation has a critical limitation: The ablation margin cannot be visualized or monitored by CT. Thus, the ablation of vertebral bone tumors can cause intra- or post-procedural injury to the spinal cord within the ablation zone (7).

Previous studies have confirmed that iodine-125 (^125^I) brachytherapy has advantages in regard to disease control in patients with solid tumors (8, 9). Thus, ^125^I brachytherapy might be effective in bone pain palliation in patients with breast cancer after the failure of EBRT; however, few reports have evaluated ^125^I brachytherapy in these patients. The purpose of our study was to evaluate the feasibility and clinical value of CT-guided ^125^I brachytherapy for pain palliation in patients with bone metastases from breast cancer.

## Materials and Methods

### Cohort and Sample Selection

In this retrospective study, we used data from patients with breast cancer and bone metastases who experienced moderate-to-severe pain. The radiological assessment of bone metastases includes enhanced CT, PET/CT, and radionuclide bone imaging. There were two groups in this study. Group A (*n* = 42) was treated with both ^125^I brachytherapy and bisphosphonates, and Group B (*n* = 48) was treated with bisphosphonates alone. This study was approved by The Ethics Committee of the Guangdong Provincial People's Hospital.

All patients in the study underwent attempts to manage their pain using chemotherapy, endocrine therapy, molecular-targeted therapy, or EBRT but did not achieve satisfactory pain relief. All enrolled patients met the following criteria: (a) underwent resection of primary tumors, which had been histologically diagnosed as breast cancer; (b) bone metastases were the only possible cause of pain; (c) score of at least four points on the “worst pain” item of the Brief Pain Inventory (BPI) in the 24 h prior to completing the inventory (10); (d) four or fewer metastatic lesions with diameter ≤5 cm; (e) expected lifespan ≥3 months; (f) the Karnofsky Performance Status Scale ≥50 (11); (g) no severe coagulation disorder [prothrombin activity <40% or platelet count <5 × 10(9)/L]; and (h) the absence of spinal cord compression or impending fracture. Additionally, all patients had received high doses of analgesics for a period of more than 1 month.

### ^125^I Seed

The radioactive ^125^I seeds (Yunke Pharmaceutical, Sichuan, China) were cylindrical titanium packages being 0.8 mm in diameter and 4.5 mm in length. The central source of the particle was an ^125^I radionuclide silver rod with a diameter of 0.5 mm and length of 3.0 mm. The matched peripheral dose was 100–140 Gy, and the average energy was 27–35 KeV. The radius of effective antitumor activity was 1.7 cm. Each seed had an initial activity of 0.0296 Gbq and a half-life of 59.6 days; about 93–97% of the brachytherapy dose was delivered within 3–5 months.

### ^125^I Brachytherapy

**Figure 1:**
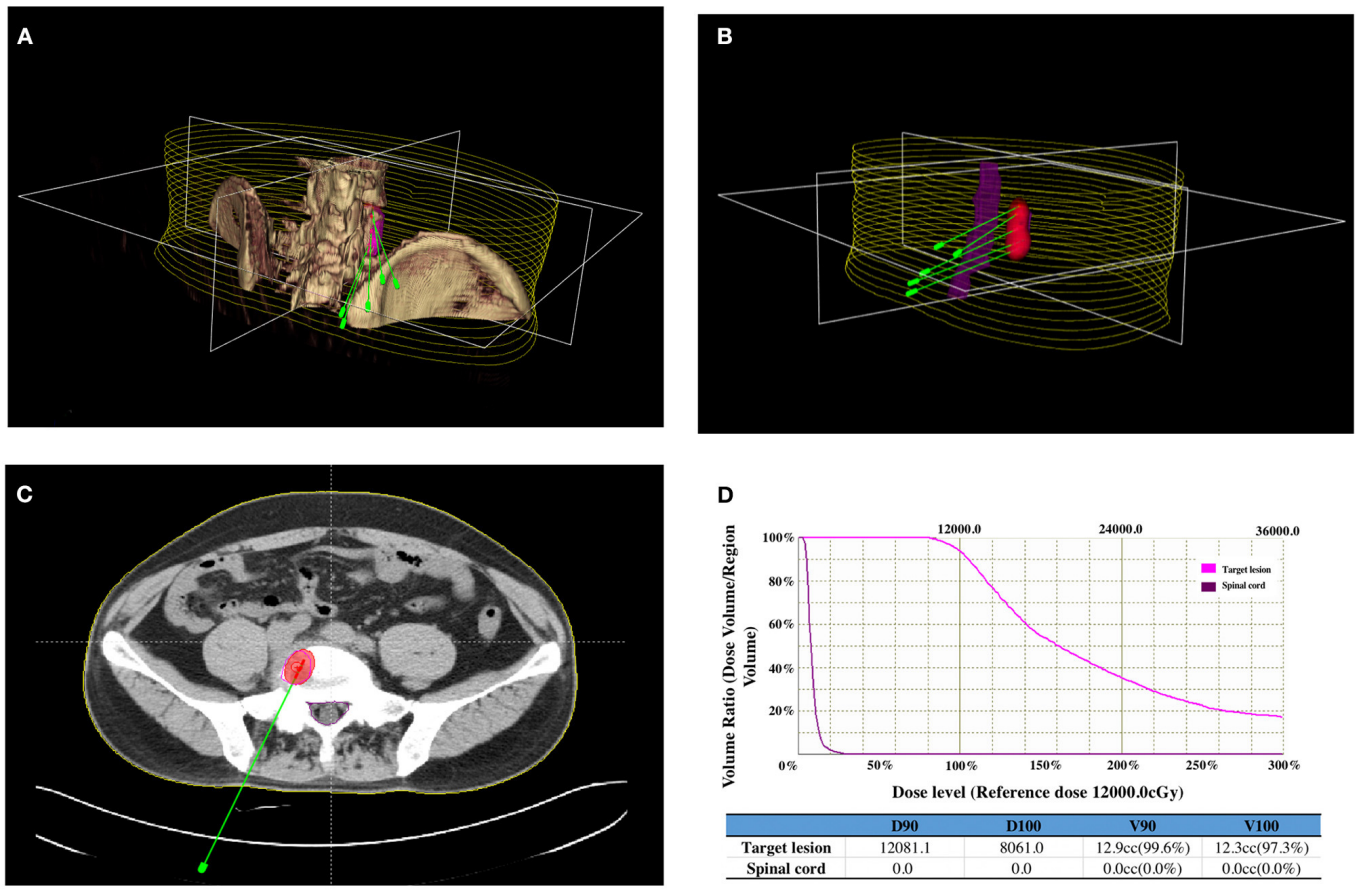
Iodine-125 (^125^I) brachytherapy treatment plan for bone metastases from breast cancer: **(A)** CT images of the lesion were reconstructed using a computerized treatment planning system (TPS), and the position of the brachytherapy applicators was calculated. **(B)** Three-dimensional image showing the relationship between the positions of the applicators, lesions, and spinal cord. **(C)** Two-dimensional image showing the irradiation dose (red area) of the ^125^I brachytherapy field, tumor contour (purple line), and spinal cord (green line). **(D)** Dose-volume histogram indicating the prescribed dose to the lesion and the dose received by the spinal cord.

Before ^125^I seed implantation, 5-mm thick CT sections were obtained for all patients. A computerized treatment planning system (TPS) (BT-RSI; Beijing Atom and High Technique Industries) was used to create a treatment plan for each patient. The gross tumor volume, planned target volume (PTV), and the surrounding vital organs were carefully delineated in every CT slice. PTV was defined as a 0.5–1.0 cm extension around the gross tumor volume. According to the three orthogonal diameters within the targeted tumor site and a prescribed matched peripheral dose, the TPS calculated the position of the brachytherapy applicator and the number of seeds to be implanted (Figures 1A,B); then, the TPS generated a dose-volume histogram that includes the isodose curves of different targets. According to the guideline of American Brachytherapy Society, the prescribed dose of the planned target was an average of 120 Gy (100–140 Gy) (12). The dose received by the surrounding organs was based on normal tissue constraint guidelines. The PTV edge accounted for 70–90% of the isodose curve; thus, 95% of the prescribed dose covered the PTV.

On the day of the procedure, patients were positioned on the CT gantry and were evaluated to locate the lesion site(s). Several 5-mm axial slices were obtained to delineate the upper and lower borders of the tumors. After achieving anesthesia with local infiltration of 5–15 ml of 1% lidocaine (Liduokayin; Yimin), an 18 G spinal needle (Yunke Pharmaceutical, Sichuan, China) was inserted into the farthest tumor edge, ~5 mm or less from the tumor border. A clip implant gun (Yunke Pharmaceutical, Sichuan, China) was then attached to the applicator for implantation. The ^125^I seeds were released from deep to superficial while retracting the needle and keeping adjacent ^125^I seeds at a distance of 5–15 mm. CT was performed after completing the implantation to identify any post-procedural complications and to verify that the position and intensity of the ^125^I seeds were in accordance with the TPS (Figures 1C,D). If the lesion showed insufficient radioactivity, the procedure was repeated to implant additional ^125^I seeds.

### Follow-Up and Evaluation Criteria

The BPI short-form (BPI-SF) (a validated visual analog scale with the score ranging from 0 to 10) was completed by each patient to obtain a measure of pain intensity (worst pain, least pain, average pain, and present pain) and pain interference (with the seven health-related quality of life dimensions: general activity, mood, walking, normal work, social activities, sleep quality, and life satisfaction). Patients completed the BPI- SF by the assistance of a study coordinator who was familiar with focal painful metastases. When multiple metastases were treated in one patient, the response was recorded for the painful area that was mostly treated. The BPI-SF was administered pre- and post-procedure. All patients underwent dynamic enhanced CT and clinical hematological examinations within 3–5 weeks after the procedure to evaluate the safety and efficacy of the therapy. The percentage of intra- and post-procedural complications was recorded according to the Toxicity Criteria of the Radiation Therapy Oncology Group (13).

### Statistical Analysis

Statistical analyses were performed using SPSS version 25.0 statistical software (IBM Corp.). Values are presented as the mean ± SD. We compared the characteristics of patients using the Pearson's chi-squared test. Statistical significance was set at *p* < 0.05. For pain palliation analyses, patients were analyzed according to their groups. Changes from baseline in the BPI-SF score were analyzed using the analysis of covariance with the treatment group and randomization stratification parameters as factors and the baseline value as a covariate. Pain palliation endpoints were analyzed using a step-down approach, in which the primary and secondary endpoints were tested sequentially to preserve the family-wise type I error rate using the following order: 12 weeks, 8 weeks, 4 weeks, and 3 days with the BPI-SF score (worst pain, least pain, average pain, and present pain). The BPI-SF score of worst pain at 12 weeks was defined as a primary endpoint. Each hypothesis was tested at a significance level of 0.05 (two-sided). Statistical significance at each step was required to test the next hypothesis.

## Results

### Characteristics of Patients

In Group A, a total of 45 ^125^I brachytherapy procedures were performed by experienced radiologists in 42 patients with 69 bone metastases. Thirty-nine (92.9%) of the 42 patients met the TPS criteria after the first procedure. Three patients who did not meet the TPS criteria received additional implantations. The final ^125^I brachytherapy achievement rate was 97.6% (41/42 patients), and the total number of implanted seeds was 1,410, with an average of 20 ± 6 seeds per lesion (range: 10–35). As shown in Table 1, Groups A and B did not significantly differ in any clinicopathological variables.

**Table 1:** Clinical characteristics of the patients and tumors.

**Characteristic**	**Group A**	**Group B**	***p***
	***n* = 42**	***n* = 48**	
**Age, years**			**0.517**
Mean age (y ± SD)	54 ± 7	51 ± 8	
Range	36–63	33–69	
**Site of bone metastases**			**0.770**
Thoracic/lumbar vertebra	27	31	
Iliac/ischium/pubic bones	7	9	
Rib/chest wall	5	3	
Other	3	5	
**Lesion diameter**			**0.846**
≤2	21	25	
>2, ≤4	17	20	
>4	4	3	
**Metastases numbers**			**0.969**
1	19	21	
2	20	24	
≥3	3	3	
**Type of bone metastases**			**0.970**
Osteolytic	21	23	
Osteoplastic	13	16	
Mixed	8	9	
**Radiological assessment**			**0.862**
Enhanced CT	30	33	
PET/CT	9	10	
Radionuclide bone imaging	3	5	
**History of treatment**			**0.714**
Chemotherapy alone	14	18	
Endocrine therapy alone	9	12	
Combination therapy	19	18	

*p, p-value; SD, standard deviation; PET/CT, Positron Emission Tomography-Computed Tomography*.

### Palliation of Bone Pain

#### Pre-Therapy (T_0_) Pain Evaluation

The BPI-SF score for pain intensity is summarized in Table 2. There was no statistical difference in the mean score for “worst pain,” “least pain,” “average pain,” and “present pain” at T_0_ between Groups A and B.

**Table 2:** Pain-related variables and score from different therapy strategies.

**BPI-SF scores**	**T**_****0****_		***p***	**T**_****1****_		***p***	**T**_****2****_		***p***	**T**_****3****_		***p***	**T**_****4****_		***p***
	**Group A**	**Group B**		**Group A**	**Group B**		**Group A**	**Group B**		**Group A**	**Group B**		**Group A**	**Group B**	
**Worst pain**			**0.067**			**0.021**			**0.071**			**0.197**			**0.028**
Mean scores	7 ± 0.5	7 ± 0.8		5 ± 0.4	7 ± 0.5		3 ± 0.6	4 ± 0.4		3 ± 0.2	3 ± 0.6		3 ± 0.5	6 ± 0.7	
Range	6–8	5–8		3–6	5–8		3–5	3–6		2–5	2–6		2–5	5–7	
**Least pain**			**0.131**			**0.039**			**0.191**			**0.063**			**0.015**
Mean scores	4 ± 0.7	4 ± 0.9		3 ± 0.7	5 ± 0.2		2 ± 0.5	3 ± 0.3		2 ± 0.6	3 ± 0.6		2 ± 0.4	4 ± 0.2	
Range	3–5	3–6		2–5	3–6		1–3	2–4		1–3	2–4		1–3	3–5	
**Average pain**			**0.073**			**0.017**			**0.227**			**0.243**			**0.027**
Mean scores	6 ± 0.6	6 ± 0.4		4 ± 0.4	6 ± 0.4		3 ± 0.6	4 ± 0.3		3 ± 0.5	3 ± 0.5		3 ± 0.4	5 ± 0.8	
Range	5–7	5–7		5–7	5–7		2–4	3–6		2–4	2–4		2–5	4–7	
**Present pain**			**0.158**			**0.018**			**0.338**			**0.479**			**0.011**
Mean scores	6 ± 0.3	6 ± 0.6		4 ± 0.8	6 ± 0.6		3 ± 0.3	4 ± 0.3		3 ± 0.5	3 ± 0.7		3 ± 0.5	5 ± 0.6	
Range	5–7	5–7		3–6	5–7		2–5	3–5		2–5	2–5		2–5	3–6	
**Morphine-equivalent 24-h dose**			**0.261**			**0.041**			**0.161**			**0.097**			**0.019**
Mean doses (mg)	136 ± 35	140 ± 29		91 ± 27	129 ± 21		53 ± 13	61 ± 16		31 ± 17	53 ± 15		34 ± 12	105 ± 23	
Range (mg)	100–180	100–180		65–120	95–160		35–70	40–75		30–65	35–75		30–65	75–135	

*T_0_, preoperatively; T_1_, 3 days post-procedure; T_2_, 4 weeks post-procedure; T_3_, 8 weeks post-procedure; T_4_, 12 weeks post-procedure; BPI-SF, brief pain inventory-short form, p, p-value*.

*Mean scores with standard deviation for points on “worst pain,” “least pain,” “average pain,” and “present pain.” The points were graded on a numeric scale ranging from 0 to 10*.

#### Pain Evaluation 3 Days (T_1_) After Therapy

In Group A, the score for “worst pain,” “least pain,” “average pain,” and “present pain” was decreased at 3 days after therapy. In Group B, there were no changes in the score for all pain intensities during T_0_ and T_1_. There was a significant difference in the score for “worst pain,” “least pain,” “average pain,” and “present pain” between Groups A and B at T_1_, as shown in Table 2.

#### Pain Evaluation 4 Weeks (T_2_), 8 Weeks (T_3_), and 12 Weeks (T_4_) After Therapy

In comparison with T_0_, the score for “worst pain,” “present pain,” “average pain,” and “least pain” was falling from T_2_ to T_4_ in Groups A and B. Regarding the pattern of pain remission, Group A achieved a continuous decline in the score for all pain intensity indices from T_2_ to T_4_, whereas Group B showed a reduction in the score from T_2_ to T_3_ and then rebounded at T_4_. There were statistical differences in the score for all pain intensity indices between Groups A and B at T_4_, and no statistical differences in the score for all pain intensity indices between Groups A and B at T_2_ and T_3_, as shown in Table 2.

#### Prescribed Dose and Adverse Reactions to Analgesics

The prescribed dose (morphine-equivalent 24-h dose) of analgesics is shown in Table 2. At T_0_, there was no significant difference in the baseline of mean morphine-equivalent 24-h dose between Groups A and B (*p* = 0.261). In addition to patients experiencing pain relief, the mean morphine-equivalent 24-h dose of Group A was decreased gradually from T_0_ to T_4_, whereas that of Group B was decreased from T_0_ to T_3_ and rebounded at T_4_. In both groups, there were statistical differences in the mean morphine-equivalent 24-h dose at T_1_ and T_4_, but no statistical differences in the mean morphine-equivalent 24-h dose at T_2_ and T_3_.

The incidence of adverse reactions (AEs) to analgesics is summarized in Table 3. As patients in Groups A and B received different dosages of analgesics, the incidence of AE or serious AE (SAE) in Group A was significantly lower than that of in Group B.

**Table 3:** Adverse reactions to analgesics from different therapy strategies.

**Adverse reactions to analgesics**	**Group A**		**Group B**	
	**AE**	**SAE**	**AE**	**SAE**
**Fatigue**				
	8 (19)	7 (17)	17 (35)	15 (31)
**Nausea**				
	9 (21)	6 (14)	15 (31)	13 (27)
**Constipation**				
	8 (19)	5 (12)	20 (42)	14 (29)
**Vomiting**				
	6 (14)	3 (7)	16 (33)	10 (21)
**Dizzy**				
	7 (17)	6 (14)	19 (40)	16 (33)

*AE, adverse event; SAE, serious adverse event*.

*Data are shown as n (%)*.

#### Domains of Interference by Pain

The score for interference by bone pain in daily life is summarized in Table 4. In addition to patients experiencing pain relief, the score on general activity, mood, sleep, normal work, and enjoyment of life was increased in both groups. However, the score for all domains shows a significant greater improvement in Group A as compared to Group B.

**Table 4:** Pain interference-related variables from different therapy strategies.

**Domains of interference by pain**	**Group A**			**Group B**			***p***
	**BT**	**AT**	**IM**	**BT**	**AT**	**IM**	
General activity	61 ± 15	79 ± 9	19 ± 10	59 ± 13	64 ± 11	7 ± 4	0.023
Mood	57 ± 9	80 ± 12	25 ± 7	61 ± 10	70 ± 9	8 ± 5	0.017
Sleep	51 ± 13	77 ± 15	28 ± 11	54 ± 7	69 ± 13	11 ± 7	0.039
Normal work	62 ± 7	85 ± 11	24 ± 7	59 ± 11	71 ± 9	9 ± 6	0.014
Enjoyment of life	59 ± 11	76 ± 13	19 ± 10	61 ± 12	69 ± 9	6 ± 3	0.027

*BT, before treatment; AT, after treatment; IM, improvement; p, p-value*.

*Mean scores with standard deviation for pain interference as “general activity,” “mood,” “sleep,” “normal work,” and “enjoyment of life.” Pain interference scores were graded on a numeric scale ranging from 0 to 100*.

#### Procedure-Related Complications

Procedure-related complications are summarized in Table 5. In Group A, the incidence of radiodermatitis, wound infection, and subcutaneous hematomas was 10, 5, and 14%, respectively. In four patients (10%), minor displacement of the ^125^I seeds was found after tumor volume shrinkage. No severe complications, such as massive bleeding and vital organ radiation injury, occurred after ^125^I brachytherapy.

**Table 5:** ^125^I brachytherapy-related complications.

**Complication**	**Group A (*****n*** **= 42)**	
	***n***	**%**
Radiodermatitis	4	10
Wound infection	2	5
Small amount of subcutaneous haematoma	6	14
Massive bleeding	0	0
Minor displacement of ^125^I seed	4	10
Vital organ radiation injures	0	0

## Discussion

Bone pain is the most common clinical symptom in patients with breast cancer and bone metastases (14). According to National Comprehensive Cancer Network guidelines, the aim of the treatment for these advanced stage patients is symptom relief rather than complete disease eradication (1).

Regional therapies for relieving pain are indicated. In clinical practice, radiotherapy due to its minimal invasion is preferred to surgical resection. A prospective clinical trial of 1,016 patients performed by the Radiation Therapy Oncology Group showed that single and multiple EBRT fractions provided equal palliation of bone pain caused by metastatic lesions and also showed that 53 and 83% of patients achieved complete pain relief and partial relief, respectively, with durable responses (ranging from 12 to 28 weeks) (15). Generally, the EBRT is promising in regard to pain palliation for most patients with breast cancer and bone metastases (16); however, in patients with a high burden of lesions and with unclear margins relative to vital organs, the role of EBRT in pain palliation might be compromised because of its less than optimal radiation dose to the lesion(s) (17). For patients with relapsing pain after receiving EBRT, bisphosphonates and personalized dosages of analgesics show the benefits in pain control, but the long-term effect was not satisfactory due to dose-related adverse effects.

In the present study, the main findings were that ^125^I brachytherapy in combination with bisphosphonates provided more efficient pain control than bisphosphonates alone in patients with breast cancer and bone metastases after external beam radiotherapy failure. After 3 days of treatment, Group A showed a significant greater decrease in visual analog scale score for pain intensity as compared to Group B, especially for “worst pain,” “worst on average,” and “present pain.” We suggest that this may be because ^125^I seeds provide a cytocidal effect without causing radioedema by continuously emitting low doses of X- and γ-rays (18). During the study, we found that patients who received ^125^I brachytherapy in combination with bisphosphonates achieved up to 12 weeks of pain control, even with lower doses of analgesics. This result may be related to the fact that ^125^I seeds have a long half-life of 59.6 days and could deliver 110–160 Gy during decay (12). Because the irradiation diameter of the ^125^I seeds is 1.7 cm, the surrounding vital organs received a less than lethal dose of irradiation, even when the prescribed dose of ^125^I brachytherapy was up to 160 Gy (19). Our findings suggested that ^125^I brachytherapy might play a good role as ablation therapy, analogous to stereotactic ablative radiotherapy (20), and our results showed that the incidence of analgesic-related adverse events of Group A were significantly lower than that of Group B. As a result, patients experienced a better quality of life and could be well-treated as close to home as possible. At the end of follow-up, we observed the pain recurrence mainly due to the attenuation of ^125^I radiation. In this case, the second ^125^I brachytherapy should be considered. ^125^I brachytherapy was a feasible treatment modality for bone metastases in this study, with a success rate of up to 97.6%. According to disease progression, we could implant the ^125^I seeds to the same or other lesions after the first treatment cycle without increasing the risk of complications (21). In this study, TPS was used to plan ^125^I seed implantation in accordance with the American Brachytherapy Society guidelines (more than 95% of the tumor receives 100% of the prescribed dose) (12). During the procedure, CT guidance clearly showed the implant volume and location of vital organs, allowing ^125^I seed implantation to be as accurate as planned. However, we found that it was not easy to manually puncture osteoplastic lesions with brachytherapy applicators, which decreased the accuracy of ^125^I seed implantation and increased the intensity of intra-procedure pain. We believe that a robotic technique for ^125^I seed implantation would improve the procedural performance.

How do our findings affect clinical practice? We found that ^125^I brachytherapy could achieve 12-week pain control and a high quality of life in patients with breast cancer and bone metastases after the failure of the EBRT. When the pain recurrence due to the attenuation of ^125^I radiation, we could repeat ^125^I brachytherapy without technique difficulty, which implies that the currently available approaches probably have the cytocidal effect in bone metastases, leading to the regression of pain. To draw definite conclusions, the mechanism of ^125^I seed on killing bone metastases cancer cell to relieve pain is needed. In addition, other reported studies on ^125^I brachytherapy have an advantage in local control for different malignant tumors (8, 9). We did not observe a significant lesion progression after 12-week post-^125^I brachytherapy (data not shown). This may be explained by the modality of radiation release of ^125^I seed. We should argue the role of ^125^I brachytherapy in the regional control of bone metastases and the prevention of SREs in a long-term follow-up study.

Our findings should be considered in the context of the limitations of this study. This was a retrospective study with a small sample size. Large sample sizes are needed to confirm our results. Further studies should also aim to study the optimal dose of ^125^I brachytherapy and the clinical benefit of treatment in regard to the prevention of SRE, progression-free survival, and overall survival.

In conclusion, the results of our study demonstrated the effectiveness of CT-guided ^125^I brachytherapy in pain palliation for patients with breast cancer and bone metastases after the failure of the EBRT. After CT-guided ^125^I brachytherapy, patients achieved a 12-week extension in pain palliation, which resulted in less analgesic consumption, shorter hospitalization, and a better quality of life.

## Conclusion

CT-guided ^125^I brachytherapy is a feasible and an effective treatment for the palliation of pain caused by bone metastases from breast cancer after external beam radiotherapy failure.

## Data Availability Statement

The raw data supporting the conclusions of this article will be made available by the authors, without undue reservation.

## Ethics Statement

The Ethics Committee of the Guangdong Provincial People's Hospital approved our study.

## Author Contributions

JH, QM, and XC conceived and designed the study. JH, XC, and ZM performed all of the ^125^I brachytherapy procedures. JH, FY, and QM analyzed the data and drafted the manuscript. WZ, QG, and ZZ were the study coordinators. RX and WZ constructed the ^125^I brachytherapy treatment plans. All authors read and approved the final manuscript.

## Conflict of Interest

The authors declare that the research was conducted in the absence of any commercial or financial relationships that could be construed as a potential conflict of interest.

## Abbreviations

^125^I: iodine-125
BPI-SF: brief pain inventory-short form
CT: computed tomography
PTV: planned target volume
SREs: skeletal-related events
TPS: treatment planning system
PET/CT: positron emission tomography-computed tomography.
